# Redefining radiotherapy for early-stage breast cancer with single dose ablative treatment: a study protocol

**DOI:** 10.1186/s12885-017-3144-5

**Published:** 2017-03-09

**Authors:** R. K. Charaghvandi, B. van Asselen, M. E. P. Philippens, H. M. Verkooijen, C. H. van Gils, P. J. van Diest, R. M. Pijnappel, M. G. G. Hobbelink, A. J. Witkamp, T. van Dalen, E. van der Wall, T. C. van Heijst, R. Koelemij, M. van Vulpen, H. J. G. D. van den Bongard

**Affiliations:** 1 Department of Radiation Oncology, University Medical Center Utrecht, Utrecht, The Netherlands; 2 Imaging Division, University Medical Center Utrecht, Utrecht, The Netherlands; 3 Julius Center, University Medical Center Utrecht, Utrecht, The Netherlands; 4 Department of Pathology, University Medical Center Utrecht, Utrecht, The Netherlands; 5 Department of Radiology and Nuclear Medicine, University Medical Center Utrecht, Utrecht, The Netherlands; 6 Department of Surgery, University Medical Center Utrecht, Utrecht, The Netherlands; 7 Department of Surgery, Diakonessenhuis, Utrecht, The Netherlands; 8 Department of Medical Oncology, University Medical Center Utrecht, Utrecht, The Netherlands; 9 Department of Surgery, St. Antonius, Nieuwegein, The Netherlands

**Keywords:** Breast cancer, Ablative, Radiosurgery, MRI-guidance, Partial breast irradiation

## Abstract

**Background:**

A shift towards less burdening and more patient friendly treatments for breast cancer is currently ongoing. In low-risk patients with early-stage disease, accelerated partial breast irradiation (APBI) is an alternative for whole breast irradiation following breast-conserving surgery. MRI-guided single dose ablative APBI has the potential to offer a minimally burdening, non-invasive treatment that could replace current breast-conserving therapy.

**Methods:**

The ABLATIVE study is a prospective, single arm, multicenter study evaluating preoperative, single dose, ablative radiation treatment in patients with early-stage breast cancer. Patients with core biopsy proven non-lobular invasive breast cancer, (estrogen receptor positive, Her2 negative, maximum tumor size 3.0 cm on diagnostic MRI) and a negative sentinel node biopsy are eligible. Radiotherapy (RT) planning will be performed using a contrast enhanced (CE) planning CT-scan, co-registered with a CE-MRI, both in supine RT position. A total of twenty-five consecutive patients will be treated with a single ablative RT dose of 20 Gy to the tumor and 15 Gy to the tumorbed. Follow-up MRIs are scheduled within 1 week, 2, 4 and 6 months after single-dose RT. Breast-conserving surgery is scheduled at six months following RT.

Primary study endpoint is pathological complete response. Secondary study endpoints are the radiological response and toxicity. Furthermore, patients will fill out questionnaires on quality of life and functional status. Cosmetic outcome will be evaluated by the treating radiation oncologist, patient and ‘Breast Cancer Conservation Treatment cosmetic results’ software. Recurrence and survival rates will be assessed. The patients will be followed up to 10 years after diagnosis. If patients give additional informed consent, a biopsy and a part of the irradiated specimen will be stored at the local Biobank and used for future research on radiotherapy response associated genotyping.

**Discussion:**

The ABLATIVE study evaluates MRI-guided single dose ablative RT in patients with early-stage breast cancer, aiming at a less burdening and non-invasive alternative for current breast-conserving treatment.

**Trial registration:**

ClinicalTrials.gov registration number NCT02316561. The trial was registrated prospectively on October 10th 2014.

**Electronic supplementary material:**

The online version of this article (doi:10.1186/s12885-017-3144-5) contains supplementary material, which is available to authorized users.

## Background

In the field of breast cancer treatment a shift towards less burdening and more patient friendly therapies is currently ongoing. Breast-conserving therapy consisting of breast-conserving surgery (BSC) followed by whole breast irradiation (WBI) is the standard treatment for early-stage disease [1, 2]. The WBI benefit with respect to local recurrence and breast cancer associated mortality varies substantially, depending on clinical and tumor characteristics [3, 4].

A main shortcoming of WBI is the protracted schedule of 16 to 23 RT fractions, ranging from 3 to 5 weeks treatment duration. Since the risk of local recurrence is low, and 62 to 88% of local recurrences are found within the vicinity of the tumor bed [5, 6], accelerated partial breast irradiation (APBI) has been investigated as an alternative to WBI. APBI can deliver a higher radiation dose solely on breast tissue directly surrounding the tumor bed in a reduced treatment time [7–9]. APBI instead of WBI following breast-conserving surgery can represent a less burdening treatment, however adequate patient selection is essential. APBI, when compared to WBI, can be associated with an increased local recurrence rate in high-risk patients, without compromising regional and distance recurrence or overall survival [10–18]. In selected patients with early-stage and low-risk characteristics, APBI can be regarded as an equivalent to WBI [18, 19]. Patient eligibility guidelines for APBI have been set up by the American Society for Radiation Oncology (ASTRO) and European Society for Radiotherapy and Oncology (ESTRO) [7, 8].

Adequate delivery of APBI is critical given that RT is aimed for high-risk tissue only and not the whole breast. Target volume definition is more precise before surgical tumor removal, when compared to a post-operative approach [20]. In addition, there is with less variability in target volume delineation across radiation oncologists [20, 21]. Also, a substantial reduction in treatment volumes can be achieved with preoperative APBI when compared with post-operative APBI, possibly leading to less treatment-related toxicity [20, 22, 23].

MRI-guided single dose APBI, prior to breast-conserving surgery, has been investigated in women with early-stage and low-risk breast cancer due to its potential to minimize RT treatment duration and toxicity [24]. However, a primary ablative RT approach to the tumor, without the performance of breast-conserving surgery, may represent an additional gain for the clinical practice. As with stereotactic RT for stage I non-small-cell lung cancer [25], non-invasive, ablative RT might be feasible as definitive treatment for early-stage breast cancer. Single dose ablative APBI has the potential to decrease the burden of multiple RT fractions, and at the same time replace breast-conserving surgery for selected patients. This could offer a non-invasive and minimally burdening treatment for women with early-stage breast cancer.

A multicenter, single-arm prospective study has been initiated in The Netherlands, in order to evaluate MRI-guided single dose ablative RT as definitive treatment for early-stage breast cancer. This paper describes the study design, which assesses an ablative treatment approach following single dose MRI-guided APBI in breast cancer.

## Methods/design

### Study design

The ABLATIVE trial was initiated as a single-arm prospective interventional study at the Radiotherapy Department of the University Medical Center (UMC) Utrecht in The Netherlands and was subsequently extended to 3 regional peripheral hospitals. The purpose of the study is to evaluate the feasibility of single dose radiotherapy as definitive treatment for early-stage breast cancer. To evaluate the pathological tumor response, breast-conserving surgery is performed at 6 months after RT. The primary study endpoint is the pathological complete response (pCR) as assessed by microscopic evaluation of the excision specimen. The secondary endpoints include radiological response, toxicity, cosmetic outcome, local, regional and distant relapse rates, and disease-free and overall survival. Also, patient reported outcome measures such as quality of life, functionality, psychological symptoms and frailty are evaluated. Furthermore, if patients provide additional informed consent for Biobank purposes, future research will evaluate radiotherapy response genotyping.

### Ethical matters

This study is set-up in agreement with the Declaration of Helsinki (Fortaleza, Brazil, October 2013) and is conducted in accordance with the Dutch Medical Research Involving Human Subjects Act (http://www.ccmo.nl). The study protocol has been approved by the Medical Research Ethics Committee of the UMC Utrecht (NL46017.041.13) and has been recorded in an international trial registry (ClinicalTrial.gov: NCT02316561). The study has been approved by the Institutional Review Board of each participating institute. Written informed consent is obtained from all patients before inclusion.

### Quality assurance

Study monitoring will be carried out centrally at the UMC Utrecht, by an independent monitor contracted by the sponsor, according to national guidelines on quality control for university medical centers [26].

### Patient recruitment and selection

Women presenting at the Department of Surgery of the UMC Utrecht and Diakonessenhuis hospital in Utrecht, St. Antonius Hospital in Nieuwegein/Utrecht or Rivierenland Hospital in Tiel are eligible for inclusion after a diagnosis of invasive breast cancer. The study initially included patients at least 60 years of age with early-stage and low-risk (cT1N0Mx) invasive ductal or ductulobular breast cancer without an indication for systemic treatment according to Dutch National Guidelines. Recently, eligibility criteria were broadened to include patients from 50 years or older, and the use of endocrine treatment was permitted. Table 1 gives an overview on the inclusion and exclusion criteria, which are in concordance to the ASTRO and ESTRO guidelines for partial breast irradiation [7, 8].

**Table 1:** Overview inclusion and exclusion criteria ABLATIVE study

Inclusion	Exclusion
World Health Organization performance status 0–2	Legal incapacity
Females ≥ 50 years^b^ with cT1N0 tumor	Known BRCA gene mutation
Females ≥ 70 years with cT1-2^b^_(maximum 3 cm)_ N0 tumor	MRI contra-indication
Tumor histology as assessed on biopsy:	Previous history of ipsilateral breast surgery and impaired cosmetic outcome, as assessed by the treating surgeon or radiation-oncologist.
- Ductal or ductolobular invasive carcinoma - Estrogen receptor positivity - HER2 receptor negative	Signs of extensive ductal carcinoma in situ on mammogram or histological biopsy.
Unifocal tumor	History of breast cancer
Tumor negative sentinel node procedure	Other type of malignancy within 5 years before breast cancer diagnosis^a, b^
Adequate understanding of the Dutch language	Collagen synthesis disease

^a^For adequately treated carcinoma in situ of the cervix or basal cell carcinoma of the skin no specific time span is required. ^b^ Criterion adjusted following the amendment

The surgeon informs patients on the possibility of a study intervention evaluating single dose RT with postponed surgical treatment instead of standard of care 16–23 fractions postoperative radiotherapy. Patients interested in trial participation receive additional information from the coordinating investigator. Furthermore, these patients are referred to the radiation oncologist at the UMC Utrecht for preoperative consultation to receive information about the standard RT treatment.

### Procedures

**Fig. 1:**
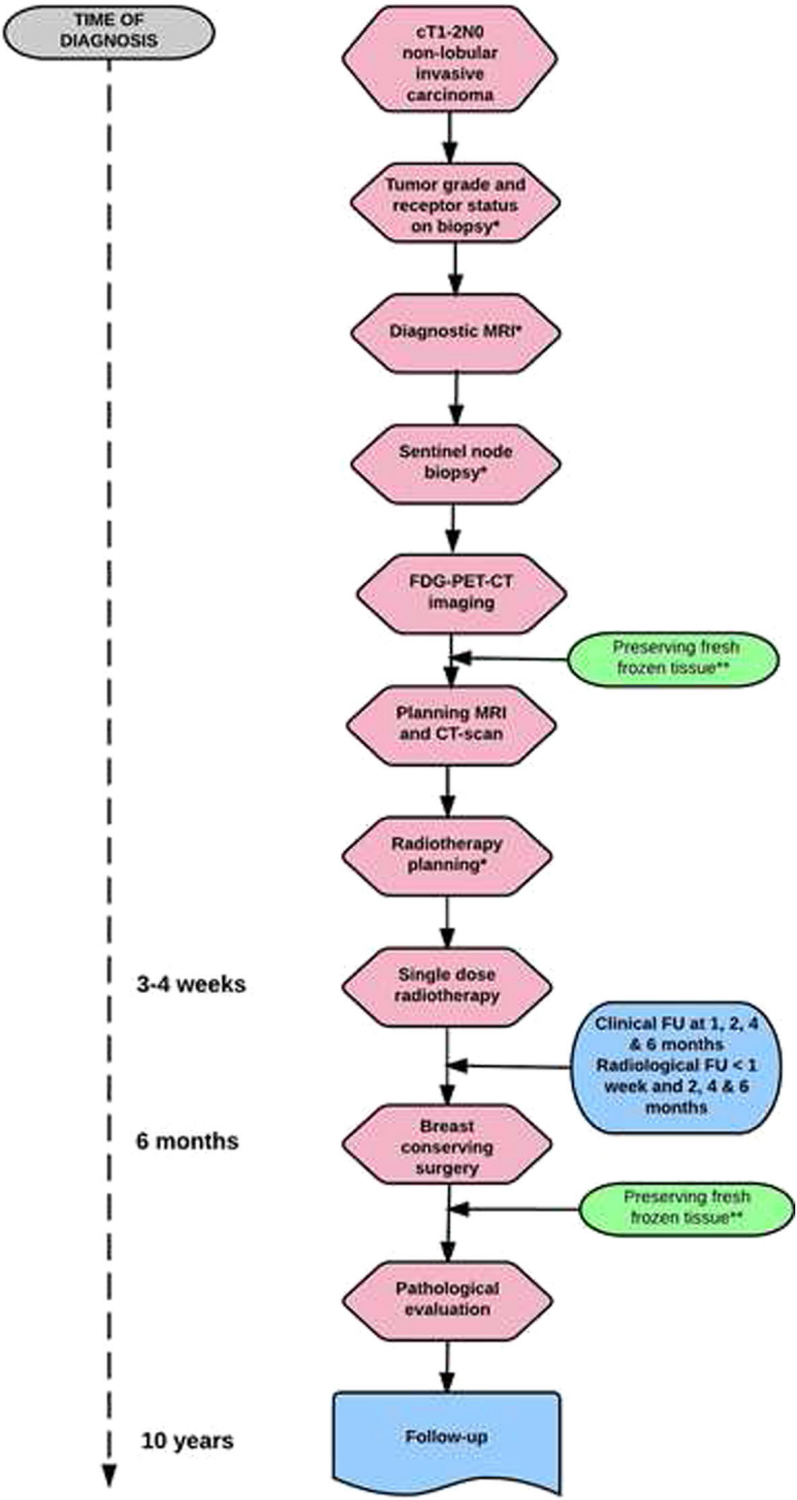
Overview study design. Legend: * reassessment eligibility criteria following procedure ** additional informed consent required

An overview of the required study procedures for single dose ablative radiotherapy is illustrated in Fig. 1. All patients will undergo the RT study procedures at the UMC Utrecht. Study patients from the participating teaching hospitals will undergo the standard of care sentinel node procedure and breast-conserving surgery in the referral hospital.

#### Diagnostic work-up

Following informed consent, eligibility criteria are assessed stepwise. First, the estrogen, progesterone and HER2 receptor, and Bloom & Richardson tumor grade are assessed on a 14-gauge histological biopsy [27]. Second, a diagnostic MRI (in prone position) is performed to assess the tumor diameter and exclude tumor multifocality or multicentricity [28]. A third step is the performance of a sentinel node procedure, using blue dye, separately from breast-conserving surgery. Only patients with a pN0 nodal status are eligible [7, 8]. For the purpose of RT response assessment, an FDG-PET-CT of the breasts is performed to acquire a baseline standard uptake value (SUV) of the tumor before irradiation.

#### Radiotherapy preparations

**Fig. 2:**
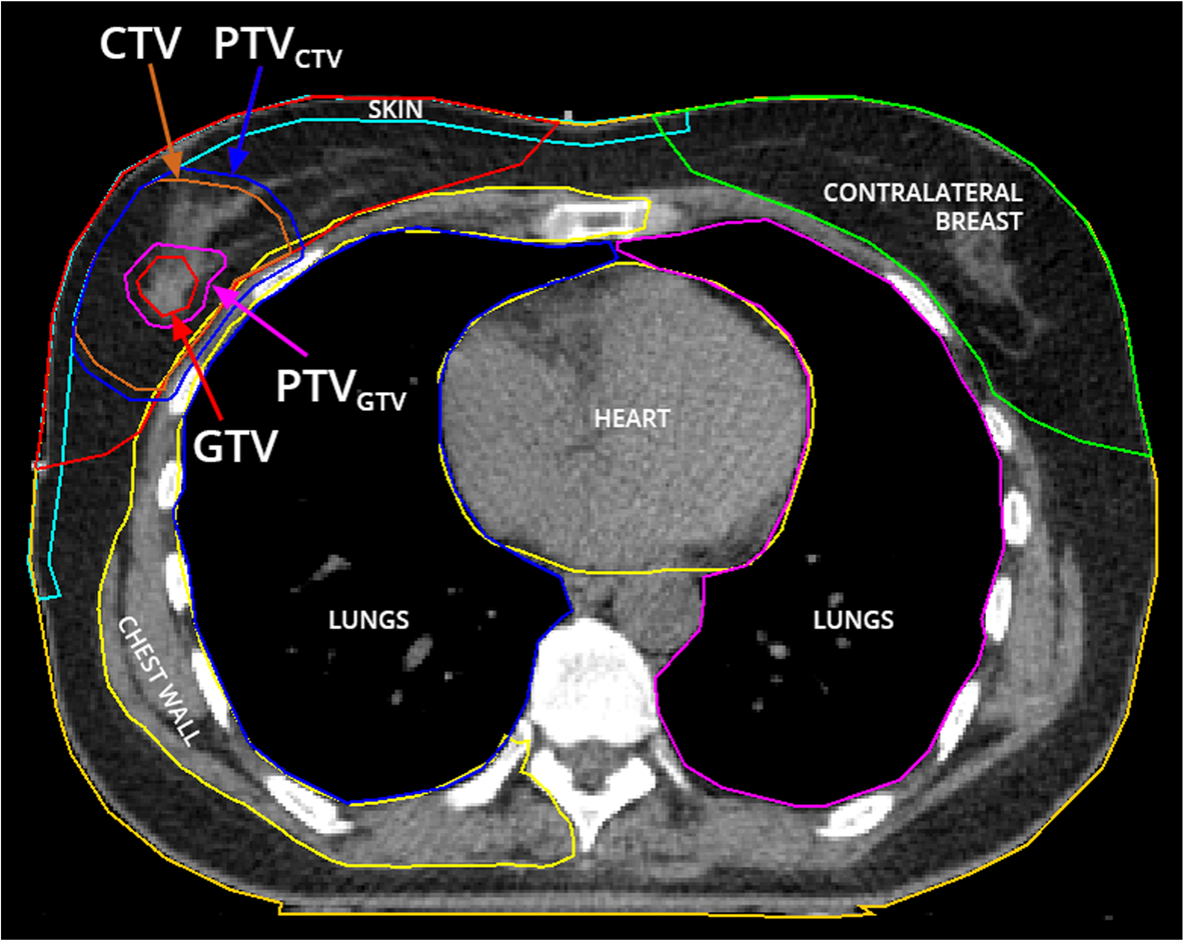
Contouring of planning target volumes and organs at risk. Legend: GTV represents the gross tumor volume, CTV the clinical target volume and PTV the planning target volume

For position verification purposes during RT delivery, an MRI compatible clip will be placed in the tumor under ultrasound guidance. For RT treatment planning, a contrast-enhanced (CE) CT-scan as well as CE and functional MRI-scan in supine RT treatment position are performed on the same day. The gross tumor volume (GTV) is delineated on CE-CT, and co-registered with the findings on CE-MRI by a radiation oncologist specialized in breast cancer. GTV delineation is verified by a dedicated breast radiologist. To account for microscopic disease, the GTV is uniformly expanded by 2 cm to create a clinical target volume (CTV), thereby excluding the first 5 mm beneath the skin and the chest wall. Both GTV and CTV are uniformly expanded by 3 mm to obtain the planning target volumes PTV_GTV_ and PTV_CTV_, respectively, thereby excluding the first 5 mm beneath the skin [29, 30]. Organs at risk (OARs), such as skin, ipsilateral and contralateral breast, lungs, heart and chest wall are delineated according to predefined protocols [29]. Figure 2 illustrates the delineations of the target volumes and OARs.

**Fig. 3:**
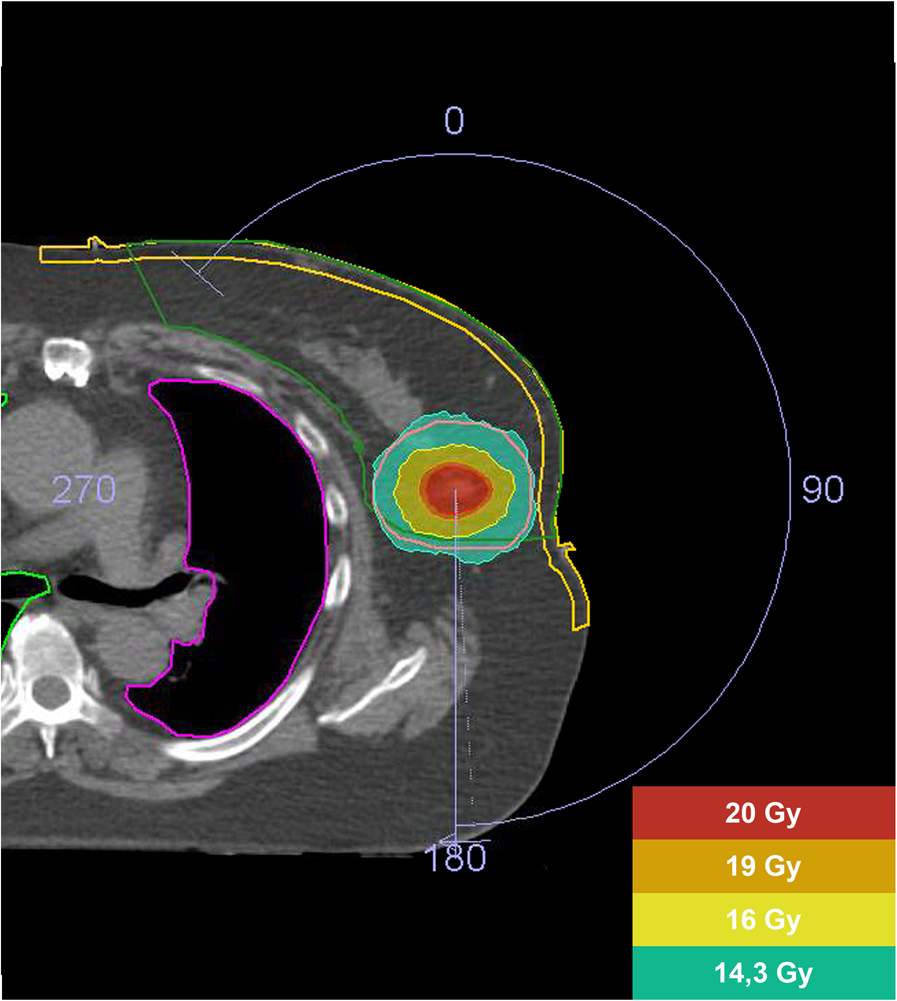
Dosimetry treatment plan single dose ablative radiotherapy. Legend: The red isodose (20 Gy) represents the prescribed dose to the gross tumor volume (GTV), the orange isodose (19 Gy) represents 95% of the prescribed dose to the GTV, the yellow isodose (16 Gy) represents 107% of the prescribed dose to the clinical target volume (CTV) and the green isodose (14.3 Gy) represents 95% of the prescribed dose to the CTV

Volumetric modulated arc therapy (VMAT) plans are created using 2 separated partial arcs, clockwise and counter clockwise. Two radiotherapy dose levels are concomitantly prescribed in one single fraction: 15 Gy to the PTV_CTV_ and 20 Gy to the PTV_GTV_. The 20 Gy single dose is equivalent to a 73.7 Gy dose in 2 Gy fractions (EQD2, α/β 4.7), resulting in a 100% 5 year tumor control probability for cT1N0 tumors [31, 32]. The single 15 Gy dose corresponds to an EQD2 of 44.1 Gy (α/β 4.7), similar to the standard hypofractionated schedule of 16 fractions of 2.66 Gy at our institution. Adequate target volume coverage is defined as 99% or more of the PTV receiving at least 95% of the prescribed dose (Fig. 2). VMAT plans are optimized for target volume coverage and a dose as low as possible to the OARs, thereby not exceeding the predefined constraints (Additional file 1) [29]. Figure 3 illustrates an example of a single dose ablative APBI treatment plan.

#### Radiotherapy treatment delivery

For position verification purposes 2 cone beam CTs (CBCT) are performed before RT delivery. The first CBCT is used for treatment position assessment, and the second one to check the tumor location after position correction. The clip in the tumor is used for position verification. Also the position of the clip relative to the chest wall is determined to quantify changes in target location or deformations. To account for intra-fraction motion, position verification and correction is performed after the first arc using a third CBCT. A last CBCT is taken after RT delivery to determine the intra-fraction motion during the second arc.

#### Follow-up after single dose ablative treatment

Following the ablative boost treatment, frequent clinical and MRI monitoring will be performed. For (early) treatment response assessment, MRIs are scheduled within 1 week and at 2, 4 and 6 months after RT. The radiologic response will be evaluated according the ‘*Response Evaluation Criteria in Solid Tumors’* guidelines [33].

#### Breast-conserving surgery

In order to assess the ablative RT effect on the tumor, breast-conserving surgery is scheduled at 6 months following RT treatment. If disease progression is clinically or radiologically suspected, surgery is performed earlier.

The surgical specimen is evaluated for radiotherapy response. Cell viability is assessed using hematoxylin and eosin staining, and additional cytokeratin 8 immunohistochemistry. The pathological response is categorized as [34]:

1. Complete pathologic response = either no residual carcinoma or no residual invasive carcinoma but DCIS may be present.
2. Partial response to therapy:
3. near complete response = minimal residual disease (<10% tumor cells)
4. evidence of response (10–50% tumor cells)
5. >50% tumor cellularity remains evident with features of response present
6. No evidence of response

The excision specimens will be revised centrally at the UMC Utrecht by one dedicated breast pathologist.

#### Follow-up

**Fig. 4:**
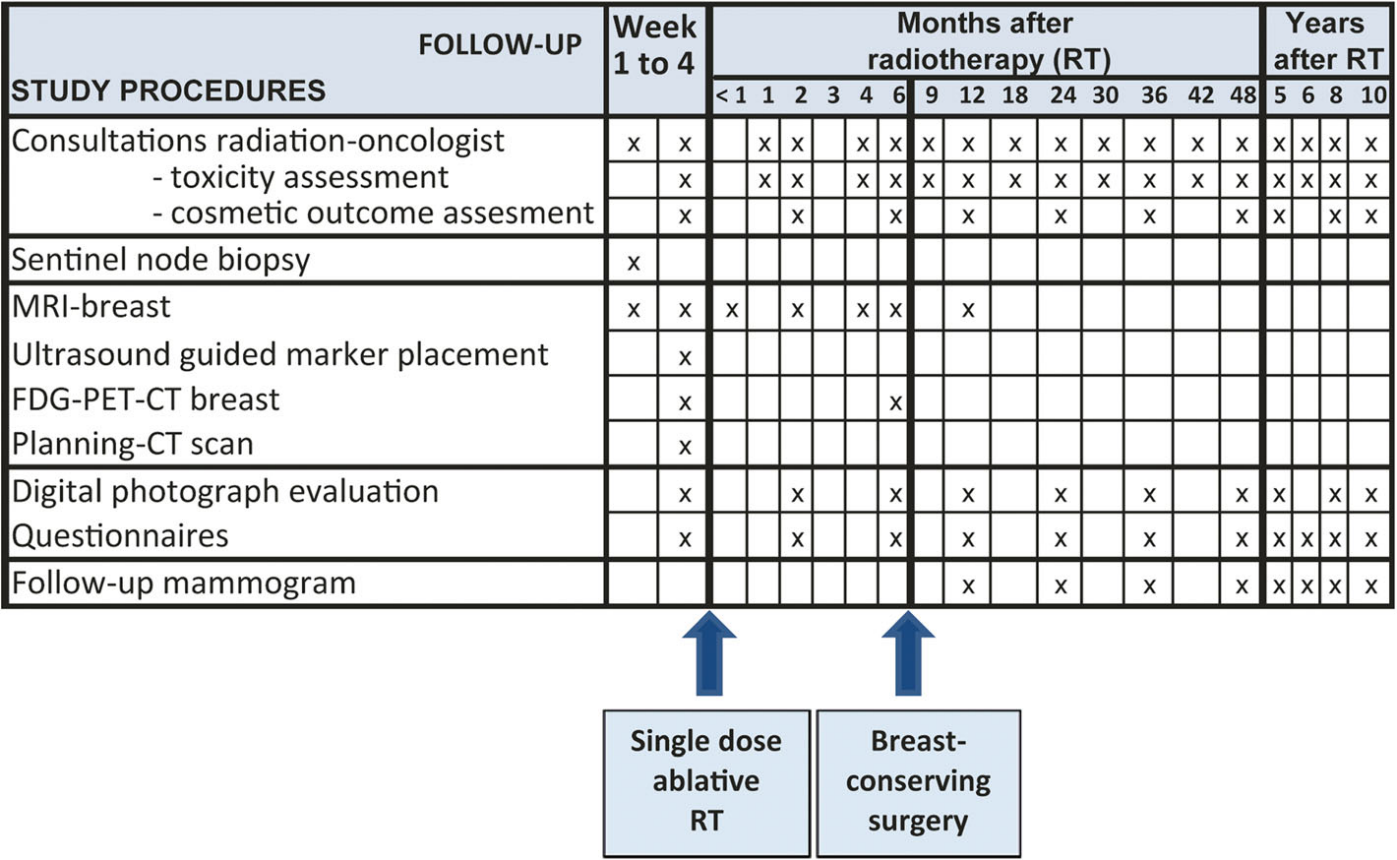
Overview study procedures and follow-up time

In our institute, follow-up visits after treatment usually consist of a yearly consultation at the outpatient department of Surgery or Radiation Oncology including mammography during 5 years. If systemic therapy is indicated, additional consultations with the medical oncologist are planned. Figure 4 illustrates the additional study procedures and follow-up time points for the study patients. Consultations with the radiation oncologist are scheduled at baseline and at predefined time points up to 10 years post diagnosis to assess long-term toxicity (Common Toxicity Criteria Adverse events version 4.03) [35]. Reporting and follow-up of (serious) adverse events is carried out according to predefined regulations of the study protocol. Cosmetic outcome is assessed (as excellent, good, fair and poor) by the radiation oncologist, thereby taking into account breast changes such as telangiectasia or fibrosis following treatment. For additional cosmetic evaluation, digital photographs of the breasts are taken and will be examined using the BCCT.core software program [36]. Patients will also fill out questionnaires on the cosmetic outcome of their breasts. Radiological follow-up will consist of yearly mammograms in the first 5 years and in the 6^th^, 8^th^ and 10^th^ year after RT. In addition, a diagnostic MRI of the breasts will be performed at 1 year after diagnosis.

##### Patient reported outcome measures

Patients are requested to fill out questionnaires on quality of life (EORTC QLQ-C30, EORTC QLQ-B23) [37], emotional symptoms (Hospital Anxiety and Depression Scale) [38], frailty (Groningen Frailty Indicator) [39] and functionality (Multidimensional Fatigue Inventory; Short Questionnaire to Assess Health enhancing Physical activity) [40, 41] at baseline and predefined time points up to 10 years after diagnosis. Furthermore, the patient’s satisfaction with the cosmetic outcome is assessed with a standardized set of questions.

##### Other procedures

Even though RT is a key modality for breast cancer treatment, no gene-expression profiles enabling a personalized treatment approach are available for the clinical practice [42]. In order to also contribute to the evolving field of radiogenomics, study patients will be required to give additional consent for a future study on radiotherapy response genotyping. For this purpose, an additional breast biopsy will be performed at baseline, and this tissue will be fresh frozen at the UMC Utrecht Biobank. Also, following breast-conserving surgery, a part of the irradiated excision specimen will be preserved.

### Sample size calculation

We expect to find a pathological complete response in 95% of the patients, as determined in the surgical specimen at six months after radiotherapy (with or without endocrine treatment). The sample size calculation is performed with the Power Analysis and Sample Size software program PASS 2008, (Hintze J, 2008. PASS 2008, NCSS, LCC. Kaysville, Utah, USA. www,ncss,com), using the exact (Clopper-Pearson) confidence interval formula in the ‘Confidence intervals for one proportion’ procedure. With an estimated pCR of 95% a sample size of 20 patients would produce a two-sided 95% confidence interval running from 75 to 100%. A total of 25 patients will be included to compensate for drop out or loss to follow-up.

### Statistical methods

The proportion of patients with pCR will be evaluated and a two-sided 95% confidence interval will be calculated. The secondary study objectives will be described without the performance of an official statistical test.

## Discussion

The ABLATIVE study is, to the best of our knowledge, the first study to evaluate the ablative effect of single dose APBI for early-stage breast cancer in current era of image-guided radiotherapy.

When pursuing a minimally burdening ablative treatment for early-stage breast cancer, external beam APBI offers the least invasive technique with the most widespread availability, when compared to brachytherapy or intraoperative techniques. A phase I dose escalation trial with 15, 18 or 21 Gy single dose RT delivered to T1 tumors has previously been evaluated in prone treatment approach [24]. Breast-conserving surgery was performed at 10 days after RT. During a median follow-up of 23 months, no dose limiting toxicity or local recurrences were observed, along with good or excellent cosmetic outcomes in all single dose APBI treated patients.

Pathological complete response following single dose APBI will be assessed by breast-conserving surgery 6 months afterwards. Local tumor control following whole breast RT only has been reported as feasible in two previous studies, performed more than two decades ago [32, 43]. In breast cancer patients with T1 carcinomas treated with a hyperfractionated 45–110 Gy schedule, the 5-year local control rates ranged from 40 up to 100% [32]. Another retrospective study evaluated hyperfractionated whole breast RT alone (median dose 76 Gy) in 319 patients with stage I-IV disease, unfit for surgery or with unresectable tumors [38]. For the total group of patients, local control rates were 56 and 44%, at 5 and 10 years follow-up, respectively. High-risk characteristics such as tumor size above 4 cm and high tumor grade were independent factors associated with local recurrence. The mean time to maximal response was 6.4 months. In another study in rectal adenocarcinoma, a time interval around 15–16 weeks after neoadjuvant chemoradiotherapy was evaluated as most adequate to result in a pathological complete response [44]). For the current study, a 6 months interval up between radiotherapy and breast-conserving surgery was considered acceptable from a patient’s perspective. Our results will show if 6 months follow-up is sufficient to enable the evaluation of a complete ablative effect. We consider a 95% pCR rate as minimum rate to take the investigational treatment to a future randomized controlled trial with breast-conserving therapy as standard treatment arm. Irrespective of the proportion of patients with a pathological complete response, the current study might have other benefits for clinical practice. A non-ablative preoperative single dose treatment is an appealing alternative to multiple postoperative RT fractions. In addition, extending the time to surgery might be considered if the treatment is not ablative at 6 months following RT. Regardless of the feasibility of an ablative treatment approach, studying preoperative RT has great potential towards a personalized and tailored RT approach. Whereas RT is an essential part of current early-stage breast cancer treatment, its effect cannot be directly evaluated since surgery is performed before WBI. For the individual patient in clinical practice, there are no predictors or biomarkers of RT response available yet. With preoperative RT, its effect can be directly investigated in the excised specimen, enabling future explorations towards RT response predictors and biomarkers.

A challenge of the current study design is setting-up the radiological follow-up between RT and surgery. The first MRI scan, performed within 1 week after RT, aims at identifying early-stage characteristics of treatment response. Data from neoadjuvant chemotherapy (NAC) for locally advanced breast cancer, have shown that the ADC values following the first cycle of NAC significantly increased from baseline, in complete and partial responders [45]. Furthermore, the previously mentioned single dose preoperative APBI study evaluated changes in CE-parameters (e.g. area under the contrast curve) and increase in ADC-values on the MRI performed at 1 week after radiotherapy. However, the clinical implications of these early MRI changes ought to be further explored [46]. Since there is no experience on RT response monitoring in breast cancer patients, additional MRIs were pragmatically scheduled at 2, 4 and 6 months following single fraction APBI. In addition, we incorporated 2 FDG-PET-CTs at baseline and preoperatively at 6 months, in order to evaluate other modalities for response assessment. MRI combined with FDG-PET-CT might have a complementary role for pCR assessment, as suggested in neoadjuvant chemotherapy studies [47]. The study results will have to identify the most useful imaging modality and time point for response assessment.

For future implementation of non-invasive ablative APBI, the performance of a separate sentinel node biopsy (SNB) has to be addressed. A SNB is performed for staging purposes only, and not with a therapeutic intent. For a select group of low-risk patients, axillary ultrasound instead of SNB might be perceived as sufficient to exclude (clinically relevant) gross nodal involvement [48], given its high specificity (range 82–98%) [49]. The impact of non-detection of micrometastases is limited, with similar survival in women with stage IA (node negative) versus stage IB disease (micrometastases) [50]. Currently, the omission of SNB is being prospectively evaluated for cT1-2 N0 disease as assessed with axillary ultrasound, treated with breast-conserving therapy [51].

At our department, the ABLATIVE study with a single dose treatment is a preparatory step towards on-line MRI-guided RT in early-stage breast cancer. The UMC Utrecht has, in collaboration with Elekta^®^ and Philips^®^, designed and prototyped the world’s first hybrid linear accelerator (MR-linac) consisting of a RT delivery system and a 1.5 Tesla MRI scanner [52]. The MR-linac provides real-time soft-tissue imaging during the actual radiation delivery [53]. Due to this targeted approach, smaller RT target volumes possibly reducing RT related toxicity, and RT dose escalation may be facilitated at the same time. The MR-linac has thus the potential to offer non-invasive, utterly precise, high-dose RT as an alternative for surgical treatment.

In conclusion, the ABLATIVE study is a multicenter prospective trial evaluating MRI-guided single dose ablative radiotherapy as definitive treatment in patients with low-risk and early-stage breast cancer.

## Additional file

Additional file 1: Overview dose prescription and constraints [29]. (JPEG 115 kb)

## Abbreviations

APBI: Accelerated partial breast irradiation
ASTRO: American Society for Radiation Oncology
BSC: Breast-conserving surgery
CBCT: Cone beam CT scan
CE: Contrast enhanced
CTV: Clinical target volume (CTV)
ESTRO: European Society for Radiotherapy and Oncology
GTV: Gross tumor volume
MR-linac: MRI linear accelerator
NAC: Neoadjuvant chemotherapy
OAR: Organ at risk
pCR: Pathological complete response
PTV_CTV_: Planning target volume of the clinical target volume
PTV_GTV_: Planning target volume of the gross tumor volume
SNB: Sentinel node biopsy
VMAT: Volumetric modulated arc therapy
WBI: Whole breast irradiation
