# Supplementary figures and images for: Redefining radiotherapy for early-stage breast cancer with single dose ablative treatment: a study protocol
Source: BMC Cancer. 2017 Mar 9;17:181. doi: 10.1186/s12885-017-3144-5 (PMC5343419; doi:10.1186/s12885-017-3144-5)

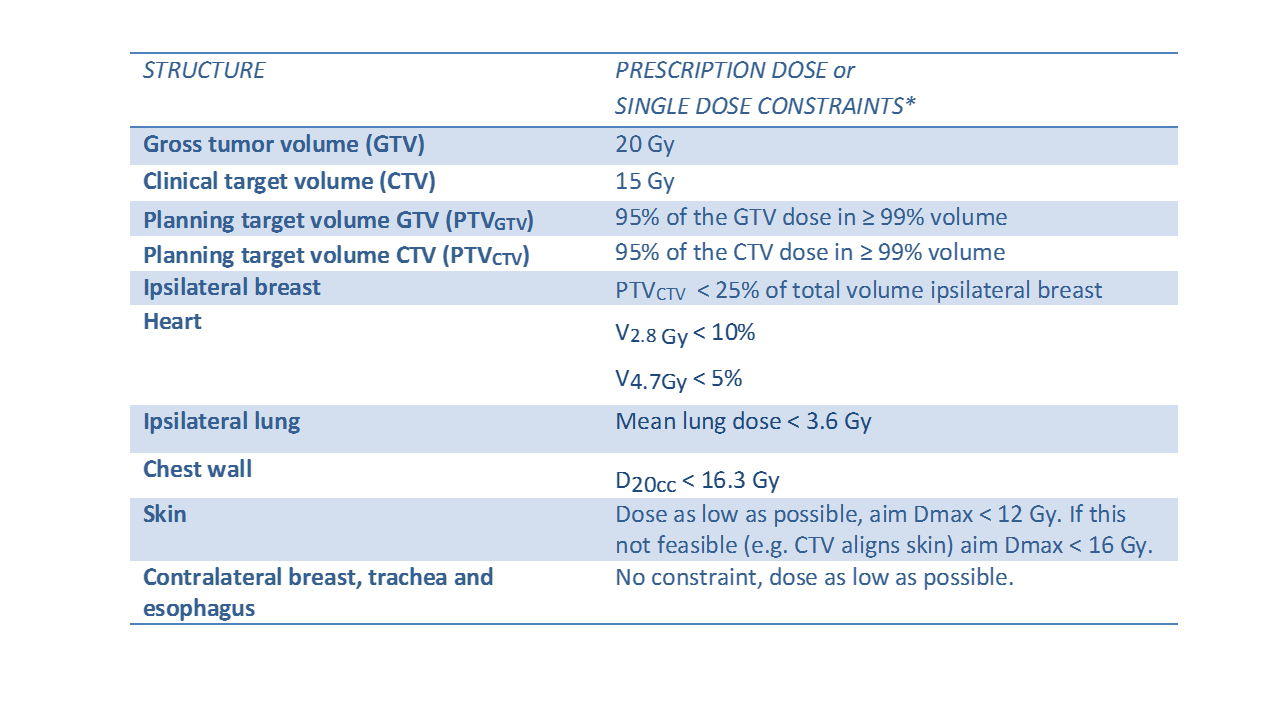

Supplement: Additional file 1: — Overview dose prescription and constraints [29]. (JPEG 115 kb) [file 12885_2017_3144_MOESM1_ESM.jpeg]
